# Understanding mental health service needs and treatment characteristics for Latin American immigrants and refugees: A focus on CBT strategies for reducing acculturative stress

**DOI:** 10.1017/s1754470x24000138

**Published:** 2024-04-11

**Authors:** Gabriela A. Nagy, Chanel Zhan, Stephanie Salcedo Rossitch, Allison M. Stafford, Andrea Mendoza, Norma F. Reyes, Rosa Gonzalez-Guarda

**Affiliations:** 1 University of Wisconsin-Milwaukee, Department of Psychology, Milwaukee, WI, USA; 2 Stanford University School of Medicine, Department of Psychiatry and Behavioral Sciences, Stanford, CA, USA; 3 Durham VA Medical Center, Durham, NC, USA; 4 Duke University School of Medicine, Department of Psychiatry and Behavioral Sciences, Durham, NC, USA; 5 Duke University School of Nursing, Durham, NC, USA; 6 University of North Carolina, Center for Health Equity Research, Chapel Hill, NC, USA

**Keywords:** Acculturative stress, Evidence-based practice, Immigrant, Latino/Hispanic, Refugee, Therapy

## Abstract

Acculturative stress is a key social driver of health impacting the mental health of immigrants and refugees from Latin America, which contributes to inequities experienced by them. While there is a robust scientific literature describing and evaluating evidence-based treatments targeting a range of psychiatric disorders, these treatments often do not primarily target acculturative stress. Thus, the present study examined how psychotherapists ought to treat acculturative stress directly in their clinical practice. Ten therapists were interviewed using a qualitative descriptive approach. Rapid contemporary content analysis was used to describe Latino/Hispanic immigrants’ most common presenting problems, the context in which they provide care for these problems, and the psychotherapeutic approaches currently utilized or considered effective in mitigating acculturative stress. Findings revealed that common mental health conditions that therapists addressed among this population, including depression, anxiety and trauma-related somatization, including the unique context in which therapy was delivered. Additionally, specific strategies for addressing acculturative stress such as the importance of acknowledging this stressor, drawing out immigration journey narratives, and behavioural activation approaches were shared. The results from this study can be used to improve the effectiveness of mental health interventions addressing acculturative stress among immigrant and refugee populations.

## Introduction

Latinos/Hispanics represent a significant portion of the United States (US) population (approximately 19.1%; 63.7 million; United States Census Bureau, n.d.), a third of which are immigrants and refugees (19.9 million; Batalova and Ward, 2023). As the number of individuals with Latin American ancestry continues to grow in the US, mental health providers will increasingly treat this demographic (Lopez and Bursztyn, 2013). The need for mental health providers to be equipped to work with this population is further underscored by inequities in stress and mental health problems among Latino/Hispanic immigrants and refugees compared with their non-Latino/Hispanic White counterparts. Despite the presence of an ‘immigrant’ or ‘nativity’ effect, whereby immigrants tend to evidence relative mental health advantages upon arrival to the US, their health declines with length of stay in the US, as well as in subsequent generations (e.g. US-born offspring; Alegría et al., 2007). Moreover, these inequities are compounded by inadequate access to mental health care (Alegría et al., 2002), as well as by the fact that a very small proportion of providers speak Spanish fluently and/or self-identify as Latino/Hispanic (i.e. 5% of psychologists; Lin et al., 2018). Therefore, it is a matter of public health to determine best practices for meeting the needs of a growing segment of the population that continues to endure an increasingly anti-immigrant landscape (Pham and Van, 2019).

The process of immigrating and acculturating to life in the US has significant health implications. During the process of *acculturation* (i.e. cultural change as a result of being in contact with another cultural context; Berry, 2006), individuals undergo a bi-directional process through which they simultaneously maintain aspects of their identity and culture, while also adopting aspects of the culture in the receiving environment, resulting in a variety of different acculturation orientations (e.g. separation, integration, marginalization and assimilation; Berry, 2003). With increased length of residence in the US and increased acculturation, Latino/Hispanic immigrants and refugees tend to adopt lifestyle behaviours that may bring about deleterious long-term impacts, such as increased smoking and tobacco use (Kondo et al., 2016) and alcohol use (Lui and Zamboanga, 2018), as well as more sedentary lifestyles and worse diet quality (Perera et al., 2020). Specifically, Latino/Hispanic immigrants and refugees whose acculturation experience is characterized by high levels of family conflict and discrimination combined with low levels of social cohesion and neighbourhood safety have higher depressive, anxiety, and substance use disorder prevalence compared with those who have more positive acculturation experiences (Roth et al., 2022).

For many Latino/Hispanic immigrants and refugees, the process of migration and acculturation to life in the US can be stressful and at times traumatic due to the presence of *acculturative stress* (i.e. the chronic and compounding challenges associated with the process of migration and acculturation). Acculturative stressors can encompass language barriers, discrimination, separation from family and family conflict, challenges securing health care, and economic and occupational stress, among others (Cervantes et al., 2016). Given the vast heterogeneity among Latino/Hispanic immigrants and refugees, there is a great deal of variability in the degree and types of stressors and trauma that can be experienced before, during and after their migration journey. Furthermore, acculturative stress is shaped by demographic characteristics such as gender (Salas-Wright et al., 2015), nationality (Cervantes et al., 2019), documentation status (Arbona et al., 2010), and socioeconomic status (Boen and Hummer, 2019), with those who are most socially disadvantaged or less legally protected tending to experience a higher proportion of acculturative stress. The intersectional nature of oppression (including xenophobia, racism, sexism, classism, ableism, and LGBTQ intolerance) not only amplifies the salience of specific facets of acculturative stress within marginalized sub-groups but also compounds the adverse social drivers of health associated with these systems of oppression. Given its role in various mental health conditions [such as depression (Driscoll and Torres, 2013), anxiety (Ornelas and Perreira, 2011; Maldonado et al., 2018), trauma-related disorders (Thibeault et al., 2017), substance use disorders (Ehlers et al., 2016), and suicidal ideation (Hovey and Magaña, 2002)], as well as physical conditions (Gonzalez-Guarda et al., 2021), it is imperative to understand the different facets of acculturative stress and closely target this type of stress in psychotherapy care when treating immigrant and refugee clients.

It is important to note as well that immigrants and refugees may face distinct facets of acculturative stress by nature of their trajectories to new environments. Often, although not in every instance, immigrants voluntarily migrate in search of better economic, education and healthcare opportunities, enhanced quality of life, and because of familial reunification. However, it is important to note that some immigrants report forced migration (i.e. ‘had to migrate’; 31% of men and 32.4% of women) and unplanned migration (43% of men and 46% of women; Torres and Wallace, 2013). In the US, immigrants can hold a range of different migration statuses including US citizen, lawful permanent resident, visa holders, and unauthorized (undocumented) immigrants. In contrast, refugees are typically forced to leave their countries of origin given inhospitable circumstances such as environmental disasters, persecution, war or violence. Individuals facing these dire circumstances benefit from legal protections (e.g. ability to legally work) that are offered to immigrants based on humanitarian grounds, such as Temporary Protected Status (TPS) and refugee status. For this reason, the findings presented herein might vary between immigrants and refugees, and across Latin American subgroups.

Despite the recognition that acculturative stress is a clinically relevant problem, there is a paucity of research on psychotherapeutic strategies that could reduce its impact, and although there is an emerging literature reporting promising outcomes across numerous psychosocial interventions addressing acculturative stress (e.g. Parra-Cardona et al., 2016; Ryan et al., 2018), their impact is precluded by lack of wide-scale dissemination. This is further compounded by limited access to mental health care experienced by Latino/Hispanic individuals (Alexandre et al., 2009). Therefore, it is an important area of enquiry to understand the strategies that therapists employ to reduce the psychological impact of immigration and adaptation for immigrants and refugees to glean helpful recommendations for other therapists aiming to support the wellbeing of this population, highlight considerations as our field moves towards developing and disseminating evidence-based treatments (EBTs) to decrease acculturative stress, and elucidate gaps to be filled by future research.

## Method

### Design

The present study aimed to characterize (a) the most common presenting problems (i.e. therapy targets) that therapists encounter when treating Latino/Hispanic immigrants and refugees; the context in which they are providing mental health care to manage acculturative stress; and the psychotherapeutic approaches currently utilized or perceived effectiveness in mitigating acculturative stress in this population. The data used in this article stem from a larger qualitative study exploring acculturative stress in immigrants and refugees from Latin America from the perspectives of immigrants and refugees, community professionals and leaders who work in sectors serving this population, and therapists, to inform the development of a psychosocial intervention to address acculturative stress in Latino/Hispanic immigrants and refugees. Herein, we report on data derived from interviews with therapists (*n* = 10). A qualitative descriptive design was employed in the study to provide a comprehensive summary of findings while retaining the language used by participants and thus its descriptive validity (Sandelowski, 2000). We utilized this method because it is a low-inference, straightforward method to characterize responses obtained from interviews.

### Sample and setting

We recruited therapists affiliated with a trusted community mental health clinic situated in the Raleigh-Durham-Chapel Hill area of North Carolina (see Supplementary material, item #1). We purposively recruited from this clinic given their focus on and expertise in supporting Spanish-speaking families in the community. Therapists were contacted via word of mouth and email. To prevent coercion of participating in the study, only those who self-referred to the study were enrolled. All individuals who initially expressed interest in participating enrolled and participated in the study. Therapists were included in the study if they (a) were a licensed therapist or trainee from a mental health discipline (e.g. clinical psychology, counselling psychology, social work, psychiatry) and (b) were affiliated with the community clinic. No therapists were excluded as long as they met inclusion criteria. Sample sizes in qualitative research vary depending on the purpose and diversity of the sample, so we recruited until data saturation (i.e. no new information arose from the interviews) was achieved.

### Procedures

The research team then determined eligibility and scheduled the interview. Therapists were compensated $50 (USD) for completing the interview. Demographic information was also collected from participants. This study was approved by the Duke University Institutional Review Board (protocol no. Pro00087792). Semi-structured interviews were conducted with therapists by the principal investigator (PI; first author). Interviews were conducted in either English or Spanish, lasted up to one hour, were audio-recorded, and were conducted either in-person at the clinic or through an online videoconferencing platform (Zoom). At the interviews, only the interviewer (PI) and the interviewee (therapist participant) were present in a private location. Each participant was only interviewed once.

The research team developed a semi-structured interview guide consisting of six broad questions followed by more specific probing questions based on the responses provided by participants. The function of the present study was to inform the development of a psychosocial intervention aimed at reducing acculturative stress in Latino/Hispanic immigrants and refugees. Therefore, the interview questions map directly onto implementation and client outcomes (e.g. appropriateness, effectiveness, adoption, satisfaction, etc.) as identified by Proctor and colleagues (2011). Additional details and the interview questions can be found in the Supplementary material (item #2).

### Study team

Our research team was a multidisciplinary team consisting of 12 people: two psychologists, one psychologist-in-training, two nurse scientists, two health services researchers, two medical students, one sociology doctoral student, one master’s in public health student, and one post-bac student. Seven (58%) team members (including the PI) identify as bilingual and bicultural Latin American, four (33%) as White American, and one (8%) as Chinese American. Most of the team (83%) identified as women. Half of the study team had prior experience with qualitative methods. The development of the codebook (template) and the coding of qualitative data was completed by seven members of this team, with direct oversight and data auditing led by the PI. Four members of the team (two nurse scientists and two health services researchers) provided consultation on the utilization of rapid qualitative methods. Several of the study team members had prior relationships with the clinic where this study was conducted in the context of prior community-academic partnered research projects as well as presentations that the PI had given at the clinic. To this end, some of the members of the research team were known to therapists who participated in this study and were adequately provided with informed consent detailing how the research team members would have access to their data.

### Data analysis

Qualitative analyses of therapist interviews used a contemporary rapid analysis approach, which allows more rapid analysis of results to promote the timely development and implementation of interventions and an understanding of the context in which an intervention will be developed (Hamilton, 2013; Hamilton, 2020). Consistent with contemporary rapid content analysis methods (e.g. Abraham et al., 2020), qualitative analysis was conducted directly from audio recordings of interviews as well as analytic memos, the latter consisting of notes and themes identified by the interviewer during and immediately following the interview. As such we did not share transcripts with participants for comment or correction. The team used respondent-by-code matrices in Microsoft Excel to facilitate data analysis. We took a hybrid approach to data analysis that incorporated deductive (i.e. using an *a priori* codebook) and inductive approaches (i.e. allowing for the inclusion of new codes arising from the data). We reached saturation with 10 participants. We did not engage in participant checking (i.e. participants were not invited to provide feedback on our findings). See Supplementary material item #3 for a step-by-step description of our approach to data analysis. Refer to Supplementary material item #4 for a description of the research team’s process for establishing credibility, verification and reflexivity (Cohen and Crabtree, 2008).

## Results

### Participants

To protect the identities of therapists (*n* = 10) enrolled in this qualitative study, we present demographic information in aggregate. Most therapists self-identified as women (*n* = 9). Half of the participants self-identified as Latino/Hispanic (*n* = 5), while the others identified as non-Latino/Hispanic White (*n* = 5). Half were born in the US (*n* = 5), and the remainder were immigrants themselves (*n* = 2) or did not provide this information (*n* = 3). All therapists were fluent English and Spanish speakers. Based on the preference of participants, all interviews were conducted in English. Their training disciplines included clinical psychology (*n* = 1), counselling psychology (*n* = 1), marriage and family therapy (*n* = 1) and social work (*n* = 7). Therapists held either master’s degrees (*n* = 9) or a doctorate (*n* = 1). Most therapists were employees at the community agency (*n* = 8) or participated in the clinic’s learning community (*n* = 2).

### Presenting problems

Therapists overwhelmingly identified depression as the most prevalent issue among their immigrant or refugee clients from Latin America. Moreover, therapists also commented on anxiety as a prominent presenting problem among clients, characterized as them ‘staying hypervigilant, feeling anxious/worried, low self-esteem’, often due to their experiences with acculturative stressors,^1^ (e.g. ‘fearing mistreatment by an employer’). Furthermore, a few therapists pointed out the prevalence of somatic symptoms (especially those identified in primary care settings), such as ‘headaches and tension’. They explained that it was often easier to address somatic complaints as this resonated better with clients compared with discussing mental problems directly. Additionally, many therapists reported that trauma occurring pre-migration (e.g. drug-related violence), during the migration journey (e.g. family separation) and post-migration (e.g. family reunification) represents a key therapy target and it was described as a ‘huge underlying current’ of a lot of the distress in clients’ lives. They described that once in the US, the community often resides in contexts that can place them at risk for trauma re-victimization:

‘They can be more vulnerable to trauma depending on the neighborhood they live in, or I hear that a lot of times immigrants and refugees are targeted because they have a lot of cash on them, not using the banking system and stuff like that.’

Therapists described how sequelae from traumatic experiences can disrupt interpersonal relationships (especially with family members) and give rise to mood, anxiety and behavioural problems in children, such as observing that their clients might report their children ‘ . . . not doing well in school, acting up, not listening, distracted’. Refer to Supplementary material item #5 for a full description of presenting problems discussed by therapists.

### The context in which therapists deliver care to address acculturative stress

Therapists described a variety of factors that contribute to the complexity of addressing acculturative stress within the context of mental health treatment, including different cultural understandings of mental health, the complexity of the US healthcare system, and the intersection of therapist and client characteristics. These contextual factors pose a range of challenges often precluding immigrants and refugees from obtaining the support they need (see Supplementary material, item #6).

#### Cultural attitudes and beliefs about mental health problems and treatment

As described by therapists, the attitudes and beliefs that clients hold about mental health problems and treatment can act as barriers to obtaining the care immigrants and refugees need to offset acculturative stressors. Therapists mentioned that their clients often have stigma and shame about having a mental health problem. One therapist summarized a common concern from clients: ‘I didn’t want to come . . . because ‘*no soy loco*’ (I’m not crazy)’. They also noted that clients hold misconceptions about the structure and purpose of psychotherapy, which represent a significant barrier to initially seeking services, although may not impede therapy itself once clients are engaged. As described by a therapist: ‘I think there is an opinion that you have to have essentially severe persistent mental illness like a psychotic disorder to necessitate psychological health [treatment]’. Another therapist mentioned: ‘ . . . [it is] difficult for many people who immigrate here and do not know what resources/help they can get’. In contrast to psychotherapy, providers observed that clients appear to be consistently hesitant about taking psychiatric medications, even when therapists themselves recommend a consultation with a psychiatric medication provider.

Aside from personally held attitudes and beliefs, therapists described that their clients care what their families and social networks think of mental health problems and treatment. Families holding positive attitudes towards seeking treatment can act as a facilitator. One therapist remarked: ‘Some people have their parents or their partners encouraging them to come and sometimes they’ll bring them’. In turn, therapists described that once their clients move past their hesitancy about engaging in treatment, they are often the ones encouraging their family and friends to seek treatment themselves.

#### Access to mental health care

Compounding the effect of acculturative stress, lack of access to mental health care represents a barrier to meeting the mental health needs of this population. Once they are interested in seeking treatment, long waitlists can be a deterrent. A therapist remarked: ‘Sometimes it’s like two months, and that does not feel good in welcoming someone . . . Otherwise, people [clients] are like, I waited too long . . . I was really into it, but then I waited too long’. Additionally, lack of knowledge securing treatment combined with language barriers (which is a facet of acculturative stress) was described as an obstacle because clients have difficulty communicating, and knowing where to look for resources and when/where to reach out for support. Furthermore, the lack of bilingual providers who can prescribe medication was also cited by a couple of therapists as a barrier to clients’ abilities to receive adequate mental health care. One therapist, for example, did not ‘know of any provider who is prescribing medicine that is bilingual . . . it seems like it is much easier to get a clinician or mental health provider who can do the assessment’.

#### Therapist–client match/mismatch

Therapists commented on the impact of the match (and mismatch) with their client based on ethnicity (e.g. Latino/Hispanic/Hispanic *vs* non-Latino/Hispanic/Hispanic) and race (e.g. White, Black, etc.). Latino/Hispanic providers commented that rapport can be enhanced by sharing Latino/Hispanic/Hispanic ancestry. To this end, a therapist noted: ‘I think it is easier to gain their trust if they know you come from a similar background’. Echoing this sentiment, another Latino/Hispanic provider remarked ‘ . . . I have a lot of family or personal experience with similar situations’. In contrast, not sharing this background can prolong the time it can take to develop rapport. A non-Latino/Hispanic, White therapist expressed:

‘I think there’s a dynamic here that may be different if a Latino/Hispanic person were working with the client, I am imagining. I am a White woman, and I don’t know how that plays out, but I often find that maybe it takes 2 sessions, 3 sessions, or even up to 10 sessions before someone [a client] shares “you know what, this is hard for me. Being here is hard” or “I am still adjusting to this. I am experiencing discrimination”.’

### Trust/mistrust

Therapists described differences in the degree of trust or mistrust that clients exhibit, which has implications for client engagement in psychotherapy. At one end of the spectrum, therapists described that there are clients who are quickly ‘jumping in’ to therapy. However, more commonly, clients’ mistrust towards therapists acts as a barrier to therapy, which therapists remarked often stems from ‘cultural beliefs around if you are going through something you keep that to yourself’ as well as fear that ‘they will be treated poorly or get in trouble if they ask for help [due to their undocumented status]’. Additionally, therapists mentioned that trust-building can be hampered by therapists invalidating/discounting their clients’ beliefs, behaviours or traditions (e.g. alternative medicine, ordering antibiotics from their home country, seeking dietary supplements to improve emotional wellbeing). Moreover, ‘building confidence and trust’ has been described as a key component of the psychotherapy process, which is especially important to keep in mind when working with individuals who are socially isolated, far away from their family and friends in their country of origin, and find themselves in a new community where they do not know who they can trust.

### Therapeutic approaches

Therapeutic approaches utilized by therapists are described in detail in Supplementary material item #7. Importantly, all the strategies described by therapists fall under the umbrella of cognitive behavioural therapies (CBTs). Moreover, therapists commented on their perceptions of optimal frequency of sessions, the settings in which care ought to be delivered, and the format (e.g. individual, group).

#### Theoretical orientation

Therapists exclusively described using contemporary CBTs in their routine clinical practice, which we interpreted to mean the broad umbrella term that is used to encompass cognitive, behavioural and mindfulness-based approaches to psychotherapy. Examples of CBTs employed by therapists included trauma focused-CBT to intervene on trauma-related sequalae and dialectical behaviour therapy to address ‘generational differences’ and ‘acculturation differences’. Importantly, therapists remarked that their use of CBTs was in line with the training provided to them by the clinic.

#### Settings

When prompted to consider optimal settings to implement interventions, therapists felt that an out-patient clinic would be an appropriate location to reach clients; this could include clinics focused on mental health or primary care. In fact, one therapist remarked, ‘ . . . often, especially for like somatic symptoms, the clients are more likely to go to their doctors than to come to therapy, and so those would be places I think would capture a boarder scope’. In addition, therapists described community centres or other frequently populated locales (e.g. churches, schools, libraries, specialized clinics) as other settings to reach Latino/Hispanic immigrants and refugees. They noted that these settings could offset acculturative stressors by fostering connections among immigrants and refugees to reduce isolation and connect them to useful resources:

‘Churches are honestly more than anything I think we hear about sort of people getting plugged into . . . we get a lot of referrals from primary care and schools and so that’s a big place where that can be a first touch, where brief intervention can happen.’

#### Frequency

When queried about the ideal frequency of therapy sessions, therapists generally agreed that weekly sessions made sense: ‘individual therapy every week, group every other week, something like that, I would imagine’. However, therapists acknowledged that due to a range of barriers facing this population, meeting at that frequency would not always be possible. To this point, one therapist described, ‘A lot of our clients have appointments that get canceled, so scheduling more frequently every week means that you are still going to see them frequently enough’.

#### Format

As a key feature of acculturative stress in immigrants and refugees concerns the disruption of social networks, many therapists described that group formats may be especially helpful for this population. For example, multiple therapists discussed the benefits of group therapy, particularly with respect to fostering a sense of community: ‘I’ve seen that the individual is important but the sense of community is too . . . group therapy gives them the opportunity to not feel alone’. Specifically, peer-to-peer conversations or groups led by a trusted member of the community may be especially useful and could encompass a ‘group support model’, ‘skills [training] group’ or English language classes to offset language-related acculturative stressors. On reflecting on the promise of such a group, a therapist remarked:

‘I think it’s so powerful when somebody else who has been through something is able to speak to people who are going through it . . . it would be amazing to have a group where somebody who has gone through the pretty big bumps in the road at the beginning can say, “This is what I did, and this is how I did it, and this is where you go to get this, and this is how you talk to the school”, and almost like a mentorship of how to survive.’

However, an individual therapy format was also deemed to be helpful for this population. Namely, therapists generally felt there were important strengths of targeting acculturative stressors in an individual therapy format. For example, one therapist remarked: ‘The individual one you get the opportunity to tackle things that you may not feel comfortable tackling in a group setting . . . sometimes they are comfortable sharing in a group, other times not’. Alternatively, a few therapists emphasized the importance of having both individual and group therapy. One therapist, for example, described the utility of having group therapy to supplement individual therapy:

‘ . . . if my clients could have a combination of that group support with individual, I think that would be magic. Because a lot of what they’re experiencing, I think, is that social isolation and feeling alone and not understood and having a peer group would really, I think, supplement the individual work that we’re doing.’

#### Components

##### Validating and normalizing acculturative stress.

Therapists described validating and normalizing clients’ distress and experiences related to the process of migration, adaptation to life in a new environment, and mental health problems they experience due to acculturative stress (e.g. ‘a lot of just like affirmation of their struggle and yeah, just acknowledgment of their hardship I think is really huge’). Additionally, therapists discussed validating stigma related to seeking mental health care. For example, a therapist remarked that they explain to their clients:

‘One of the first things I do is tell them it takes courage to come [to therapy] . . . they think that people that come here are crazy, but that is not true. People that come are seeking help and it requires them to have that courage to find the help they need to live out the dreams they came to this country for.’

Therapists also noted that this validation is helpful when it comes from the therapist, as culturally they are perceived as being a credible and respected figure, and that also the benefits of having a support group could be that it might normalize multiple facets of the immigrant experience by other immigrants and refugees, such as ‘how to cope with being a wife for your family, being an immigrant, navigating a healthcare system that might not be familiar to them, trauma in migration’.

##### Psychoeducation.

Most therapists explained that they weave psychoeducation techniques (e.g. ‘ . . . a very basic introduction about what they can expect when they get connected to their mental health service’) into their therapy to increase engagement. As they described, psychoeducation could be about the process of therapy, diagnoses assigned, and the concept of acculturative stress. However, the therapists highlighted a lack of psychoeducation materials they could distribute to their clients on immigration and acculturative stress. Thus, there is a need for disseminable psychoeducation materials to help ‘immigrants know what to expect . . . [and] your children’s path for acculturation’.

##### Discussion, reflection, and drawing out narratives.

Several therapists noted the importance of providing a safe space for clients to discuss and reflect on their immigration journey and subsequent adaptation, which may at times be at odds with rigidly sticking to a concrete agenda. A therapist explained: ‘When we as therapists go in and tell them here is the agenda, here is what we are going to do, it takes away from what they really need’. This sentiment was echoed by another therapist who explained, ‘A lot of our clients are very oratory; like a worksheet or very structured intervention does not work for many of our clients’. To facilitate drawing out the clients’ narrative, therapists rely on innovative tools like asking them to ‘color what they miss from their countries’ (when working with children) and using ‘Google Earth and look up the person’s home country’. Furthermore, therapists pose a range of questions to assist in this process, such as ‘What things do you like about living in this culture?’, ‘What things don’t you like?’, ‘What kinds of things did you like about your home culture and what things didn’t you like?’. Additionally, therapists described connecting treatment components to reflecting on clients’ home country:

‘I have clients do a mindfulness exercise to help them think about their home country. For her, her face just transformed, she talked about a lot of social anxiety with trauma, and she talked about her family members who are there. I told her, this is your space, if you are having a hard time sleeping, you are always able to imagine that you are there. She left looking a bit more hopeful.’

##### Perspective-taking and cultural brokering.

Several therapists described the presence of an intergenerational gap between immigrant parents and their children, characterized by members within a family acculturating at different rates (usually parents and children) and an inability for each generation to understand one another, which generates conflict. In some cases, this is pronounced when adult immigrant parents ‘didn’t get an adolescence’ and therefore might pose questions to therapists about their adolescent children such as ‘Why are they complaining? They have everything. I didn’t have anything!’ or ‘Why are you having all these feelings and why are you having all these opinions?’. Parents and children may differ in their perspectives on topics such as dating and what to spend money on – for example, parents may want to pay for a *Quinceañ*e*ra* (coming of age party for females ages 15) versus children wanting to use that money towards college. When intergenerational gaps are mentioned in treatment, therapists described fostering perspective-taking between parents and children to ‘bridge the gap between the parent and the child’, which could entail ‘going back and forth’. Moreover, therapists described cultural brokering whereby the goal is to ‘help negotiate, improve communication’ between parents and their children. A therapist described she might say to a child client:

‘ . . . your mom is making you do all of this stuff around the house and not your brother and I can see that seems really unfair to you and she’s preparing you for the life that she thinks .. . she’s giving you the tools that she thinks will make you successful you know.’

Importantly, however, it was noted that the effectiveness of this strategy ‘depends on how receptive each side is; sometimes [the conflict] does not get resolved’.

##### Connection to community resources.

As noted by therapists, acculturative stressors are often exacerbated by either lack of connection to existing resources to meet basic needs (e.g. mental health care, emergency food assistance, intimate partner violence resources) or by challenges navigating complex systems that are not built to optimally include them (e.g. the health care sector, the education sector, the legal sector). The experience of not being connected to resources can lead clients to ‘feel isolated and alone as an immigrant’, which is especially true of clients who are ‘extremely rural without transportation, they’re mostly agricultural workers so they’re isolated geographically and socially’. Therapists work to connect clients with community resources ranging from community centres to explaining to them their rights. This strategy is so important, in fact, that one therapist noted that receiving training in being able to ‘link resources to clients’ could be more helpful than receiving additional training in psychotherapy. Additionally, connecting clients to needed resources is facilitated by having those resources in proximity, and in the case of the clinic where participants were recruited, the business on the same block included a community resource centre, a community food bank, and a thrift store where they could secure important goods cheaply or freely. Therapists noted that resourcing their clients potentially helps them connect with others and can give rise to feeling ‘you are not alone in dealing with this, feel some sort of community and commiserating with one another’. Furthermore, a therapist remarked: ‘It also touches on the collectivism in Latino/Hispanic culture, the importance of feeling connected’.

##### Therapist’s self-involved, self-disclosure.

A couple of therapists felt strongly about the effectiveness of self-disclosure in creating rapport with clients. This strategy was deemed to be effective by therapists who were themselves or were offspring of immigrants or refugees as this can help ‘gain their trust’, ‘build rapport with the clients’ and promote high levels of client engagement (e.g. fewer missed appointments). Similarly, another therapist – not an immigrant herself (non-Latino/Hispanic, White) – noted the positive impact that her self-disclosure of her political stance had on building trust with the client. She remarked:

‘I had a client who had a dad who was in the detention center, detained and awaiting trial, and I remember talking to her mom, and the mom was like “but he did deserve it, because he did break the law”. And I was like “well some laws aren’t fair or just”, and then she gave me a look, and it was a moment when she was like “oh, we can talk about this”. Me being able to share some feelings about it because it revealed some politics . . . helped them feel more comfortable.’

This comment by a therapist that we interviewed highlights that therapists need not come from the exact same background to be able to have effective self-disclosure; rather, self-disclosure could pertain to entail commonalities in identities and lived experiences, the normalization of struggles that are part of the human experience, and reactions (such as empathic understanding) about content shared by the client, for instance.

##### Behavioral strategies.

To combat some acculturative stressors, therapists mentioned helping their clients become behaviourally activated. One therapist described a strength-based approach: ‘ . . . for farmworkers and for poultry, probably for a lot of different industries, there’s very limited control over the environment. And so, choosing when to have a phone call, when to do your laundry, all these things are determined by someone else. And so, when I can help someone identify, what is one thing you can control that is in your realm of control, I think that can create some confidence and sense of empowerment’. Examples of behavioural activation assignments described by therapists included, but were not limited to, going to parks, being in nature, and scheduling time to talk to friends.

## Discussion

The present study sought to describe mental health conditions linked to acculturative stress among Latino/Hispanic immigrants and refugees from Latin America residing in the Southern US (North Carolina) and how these were addressed by therapists with expertise working with this population. The context in which this care was provided was also explored. Our findings resonate with and add to the literature in several ways.

Results from a descriptive analysis of interviews with 10 therapists identified common mental health problems including depression, anxiety, trauma and behavioural problems associated with acculturative stress. Furthermore, it is possible that individuals with untreated trauma may be more susceptible to experiencing mental health problems and acculturative stress. Therapists also discussed the unique somatization of these stressors and mental health conditions they perceived to be unique for this population. These findings coincide with existing research noting that somatization is a cultural idiom of distress that is common among Latino/Hispanic immigrants and refugees (e.g. Hulme, 1996). To ascertain the prevalence of distinct presenting problems of immigrants and refugees in routine clinical care and their associations with acculturative stress, future research ought to conduct a large-scale chart review to uncover patterns and associations with demographic and migration-related factors.

Our study provided a nuanced understanding of the kinds of barriers that Latino/Hispanic individuals face contributing to healthcare inequities. For instance, an important finding was that there can be an initial misunderstanding about what the function of psychotherapy is – that it is for people who are *loco* [crazy], or for people with high severity of illness. However, once individuals are in therapy, the internalized stigma decreases, and those individuals may influence other people to seek therapy too, providing social support and encouragement for others to attend mental health services. These findings point to the need for mental health fields to identify avenues through which they may demystify mental health conditions and mental health care, as well as reduce stigma about both. These could include psychoeducation, educational entertainment, experiential workshops (e.g. utilizing acceptance and commitment therapy techniques), and immersion activities with people living with mental health conditions, among others.

Additionally, one potential contributor to the lack of understanding and stigma about mental health treatment within the Latino/Hispanic community could be related to a legacy of structural racism that has led to treatments being developed and tested on primarily White samples, and a lack of formal focus on culture, context and systemic oppression in existing EBTs. The findings from our study highlight the need to focus on culture and context, especially for clients who have recently migrated and are navigating distinct cultural realities. Not only this, but psychotherapeutic strategies ought to also address upstream structural factors that can give rise to marginalization and chronic stress to communities of colour. Therefore, we recommend that any psychotherapeutic treatments adapted for Latino/Hispanic immigrants and refugees focus on the structures and systems that perpetuate acculturative stress as well as strategies for reducing it, recognizing that current EBTs are limited in their discussion about how to address this area. Therefore, meeting the needs of Latino/Hispanic immigrants and refugees in psychotherapy will necessitate creative and innovative psychotherapeutic approaches that are culturally, linguistically, and contextually relevant for this population, as well as engaging in rigorous adaptations of existing EBT.

One important finding in our study concerned the categories pertaining to trust/mistrust among therapists and their clients, as well as the role of therapist–client match (in other words, when a Latino/Hispanic client’s therapist was similarly Latino/Hispanic) and mismatch (when the client’s therapist was Latino/Hispanic White) in the therapeutic alliance. While our study was descriptive in nature and we did not ask therapists to describe the associations among these constructs, our analysis suggests that the perceived benefit of having a therapist who shares a client’s cultural background is not inherently necessary in the cultural match *per se*, but rather the extent to which a therapist seeks to understand their client’s cultural background and experiences with immigration, as well as the extent to which therapists provide culturally humble care. Thus, recognizing that internalized stigma about mental health conditions and care are prevalent in the Latino/Hispanic community (Nadeem et al., 2007) and the discrimination that this population may face in the health care system (Sheppard et al., 2014), it is especially important for therapists (especially those of different backgrounds from their clients) to work towards creating therapeutic environments that give rise to trust, understanding and humility between therapists and their clients. Consistent with relevant research, ‘light’ self-disclosure from the therapist can help foster more personable interactions that can allow clients to feel more comfortable and subsequently increase trust (Audet and Everall, 2010).

Furthermore, our findings suggest there may be a link between securing and maintaining trust with the client and cultural humility/competence. Namely, therapists caution against losing clients’ trust by invalidating or discounting clients’ beliefs, behaviours or traditions related to caring for their mental health (e.g. alternative medicine), and this may be partly due to the documented instances of invalidation from health care providers toward this population (Sheppard et al., 2014). Therefore, it could be helpful for therapists to proactively shore up knowledge and skills around the provision of culturally humble care to Latino/Hispanic immigrants and refugees rather than responding to an impasse or rupture when it occurs. As noted by our findings, one way to achieve this goal could be by relying on appropriate therapist self-disclosure to build rapport and trust in the therapeutic relationship. It should be noted that while therapists in this study who discussed self-disclosure noted its potential benefits to the relationship (e.g. instances where self-disclosure normalized and validated clients’ experiences with acculturative stress), some scholars have proposed that therapist self-disclosure could, at times, be exploitative, cross boundaries, and distract the focus of treatment away from the client (Peterson, 2002). Moreover, some have noted that therapist self-disclosure can involve problematic therapy interactions based on cultural assumptions (e.g. a White therapist invertedly communicates to a client of colour that their personal values are a normative reference for desirable human development and functioning; Lee, 2014). Clinicians are encouraged to seek supervision and consultation pertaining to ways to engage in appropriate self-disclosure.

Our study also adds to the literature by illustrating the techniques that therapists currently use to address acculturative stress among Latino/Hispanic immigrants and refugees, a largely unexplored topic. Therapists recommended the use of individual therapy to dive deep into specific problems and group therapy to reduce social isolation by connecting with other immigrants and refugees. Therapists described a range of strategies to reduce acculturative stress, all in line with a CBT theoretical orientation (especially behavioural strategies). Namely, therapists focus on validating their clients and normalizing their reactions to acculturative stressors they experience, while encouraging their clients to describe their experiences with immigration and subsequent adaptation. Therapists also provide psychoeducation about the process of treatment. During instances when clients are struggling with intergenerational gaps, therapists help families engage with perspective-taking and they play the role of cultural brokers, navigating between Latin American and US cultures. Considering the lack of connection that immigrants and refugees from Latin America experience, therapists spend time connecting clients with local community resources. Therapists discussed the impact of having or not having a shared Latino/Hispanic identity as well as immigrant background with their clients. When there is a match in ethnicity or immigrant status, therapists therapeutically disclose personal information that is relevant to struggles their clients might be facing in their adaptation following migration. These findings also highlight the need to tailor treatments and delivery methods to better respond to the cultural needs and preferences of Latino/Hispanic immigrants and refugees, as well as the contexts in which they reside.

### Limitations and strengths

There are several limitations to this study. First, most participants in this study were therapists from one clinic. It is possible that different perspectives would have been garnered if a different population of Latino/Hispanic immigrants and refugees were being served by this organization (e.g. rural versus urban setting). Similarly, delivery contexts of mental health would have likely been uncovered if the organization and approach to training/supervision were different. The mental health organization that serves as the main study site was a culturally specific organization for treating Latino/Hispanic immigrants and refugees, with a noted history of excellence. More needs could have been identified in organizations with less expertise in serving this population.

Despite our best efforts to recruit a wide array of opinions from therapists of distinct training disciplines, many enrolled participants were trained as social workers, and as compared with the proportion of master’s- and doctoral-level therapists hired at the clinic, we under-sampled doctorate-level clinicians (holding a PhD) and were unable to recruit psychiatrists (holding an MD). Future studies ought to work towards recruiting a wider array of therapists from whom Latino/Hispanic immigrants and refugees may seek treatment who are not included in this study, such as PhD-level counselling psychologists, PsyD-trained clinical psychologists, psychiatrists, and psychiatric mental health nurse practitioners.

Furthermore, we did not collect detailed information pertaining to the specific training and clinical experience of therapists to characterize the categories we identified, and therefore we are limited in our ability to determine associations among their expertise and the way that they provide psychotherapeutic care for this population. Relatedly, we sought to understand a large range of approaches that therapists use to reduce acculturative stress in routine clinical care; however, therapists exclusively discussed approaches aligned with a CBT theoretical approach, which could have been due to the kinds of approaches encouraged by the clinic. Additionally, it is important to note that only one therapist was male, so it is possible that gender identity influenced the lens through which therapists interacted with Latino/Hispanic clients.

Another limitation concern is that although therapists elaborated on the most common mental health conditions associated with acculturative stress, this study was not designed to examine their prevalence. We captured therapists’ perceptions of common presenting problems, rather than taking a quantitative or mixed methods approach to reviewing the presenting problems of clients served by the clinic to determine actual frequencies through a chart review; nor did the present study look at personality factors or other pre-dispositions of the immigrants. Therefore, interpretations of the prevalence of conditions should not be made.

This study was focused on impressions derived from interviews with therapists about general approaches that they take to reduce acculturative stress in Latin American immigrants and refugees who seek treatment after migration to the US, but we did not collect data about the circumstances of exit (e.g. violence, poverty, lack of educational opportunities, etc.) leading to their migration, or pre-existing mental health conditions that may have been present before migration which would have allowed us to characterize the kinds of clients served by the therapists interviewed. Notwithstanding, the exploratory qualitative approach presented herein represents a means to gain a deeper understanding of the patients served by the therapists interviewed in this study rather than to generalize findings to a broader population of immigrants and refugees from Latin America.

Lastly, the present study addresses the perceptions of the therapists. Yet the voices of the clients themselves would be an important aspect to inform intervention development. It would be useful for future research to capture what their key concerns are (i.e. targets for intervention), what they would deem useful in reducing acculturative stress, what may not be as useful, and the impact of any interventions (existing or novel) aimed at reducing acculturative stress. Moreover, considering our findings pertaining to therapist match/mismatch, trust/mistrust, and therapist self-disclosure, it would be helpful to explore clients’ perceptions of these constructs and how they may relate to treatment engagement, treatment outcomes, and the therapeutic relationship.

Despite the enumerated study limitations, there are several strengths to this study that are worth noting. The study used a rapid content analysis approach that allowed for a quicker transition between questioning and the generation of new knowledge for action. Data analysis procedures were also rigorous and included a combination of deductively applied theory-driven constructs and the inductive emergence of new constructs that informed the categories described. The inclusion of an interdisciplinary team with diverse identities, including reflexivity, added to the credibility and verification of the findings. Lastly, this study engaged diverse and experienced therapists who identified as both Latino/Hispanic and non-Latino/Hispanic with an extensive history of working with Latino/Hispanic immigrants and refugees in North Carolina, thus leveraging their collective expertise.

### Implications

There are important implications for the provision of mental health care for Latino/Hispanic immigrants and refugees. First, given the perceived influence that acculturative stress has on mental health and the somatization of psychological stressors, it is critical to address the context of adapting to US culture after immigration as part of the standard of care. Principles from the trauma-informed movement can be applied to generate an environment for more immigrant-informed mental health care that recognizes the psychological and physical health consequences of acculturative stress and for many, associated traumatic experiences. This study uncovered the importance of validating the difficulty of immigrant experiences and acculturating to new life. We know from the field of trauma-informed care about the importance of validating the psychological and physical consequences of psychological trauma. While caring for Latino/Hispanic immigrants and refugees, it is also critical to address issues of trust/mistrust, therapist–client match, and culturally specific attitudes about mental illness to eliminate barriers to accessing healthcare.

Findings from this study suggest the need to adapt current practices that focus on the therapist not revealing their own experience (often referred to as self-involved, self-disclosure in psychotherapy) to ones that allow for disclosure of relevant personal struggles with immigration or perspective of justice in immigration law as a key step in establishing a therapeutic alliance. Indeed, this study calls for a change in the paradigm of training, which has largely valued Western norms of distance between therapist and client, to one that is more culturally humble and recognizes the need for Latino/Hispanic immigrants and refugees to connect with their therapist on a personal level (i.e. aligned with the cultural value of *personalismo*).

## Supplementary Material

1

## Data Availability

Project materials can be accessed by contacting the corresponding author.

## Key learning aims

1. To explore how acculturative stress represents a key driver of mental health for immigrants and refugees from Latin America.
2. To consider ways that cognitive behavioural therapy (CBT) elements can be applied to treating acculturative stress and mental health problems among immigrants and refugees from Latin America.
3. To expand upon strategies that can be helpful in rapport-building and establishing trust with patients who are struggling with acculturative stress.

## Key practice points

1. Acculturative stress is a common phenomenon linked to mental health problems and health disparities among immigrants and refugees from Latin America.
2. Despite a dearth of evidence pertaining to how therapists can address acculturative stress in routine practice, our research found that there are several CBT elements that are well-suited for this problem including psychoeducation, skills training in the area of perspective-taking, linking patients to community resources, and behavioural strategies.
3. There are several strategies that therapists can use to build, maintain and enhance therapeutic relationships with clients such as providing validation, drawing out reflection and narrative related to migration journeys and subsequent adaption, and engaging in therapist self-disclosure.

## Footnotes

1 The term ‘acculturative stress’ was first utilized by the interviewer given a careful review of the scientific literature focused on understanding sources of stress impacting immigrants and refugee populations before, during and after migration. Despite the term being first introduced by the interviewer, most of the therapists who were interviewed for this study also utilized the term ‘acculturative stress’, indicating that it was consistent with their discourse in relation to the patients they treat.
