# Supplementary material for: Understanding mental health service needs and treatment characteristics for Latin American immigrants and refugees: A focus on CBT strategies for reducing acculturative stress
Source: Cogn Behav Therap. Author manuscript; Available in PMC 2025 Aug 22. (PMC12369657; doi:10.1017/s1754470x24000138)
Supplement: 1 [file NIHMS1979540-supplement-1.docx]

Supplementary materials #1

Contextual Details Regarding Recruitment Site

| 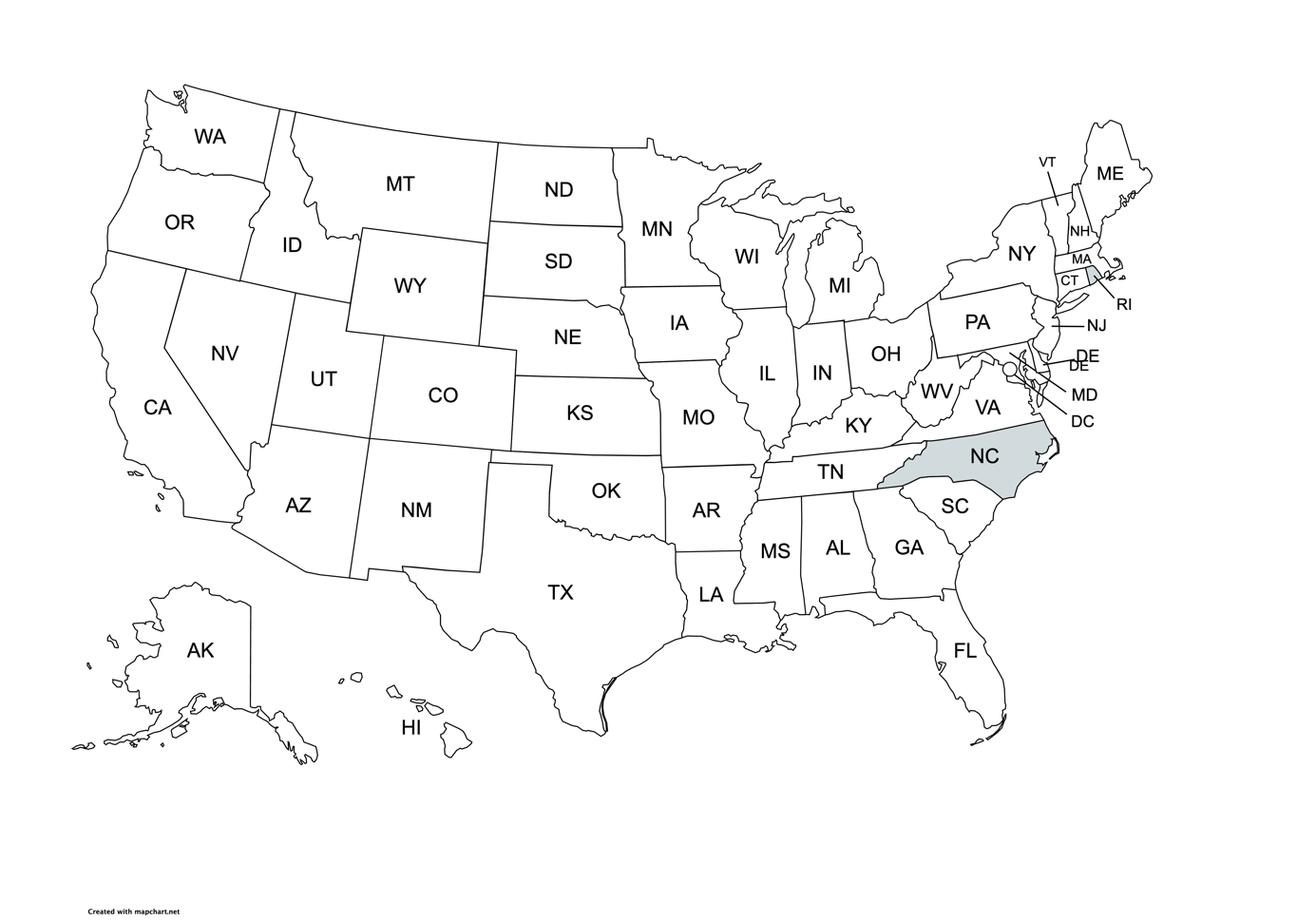 |
| --- |
| Participants (mental health therapists) were recruited from the Raleigh-Durham-Chapel Hill area of North Carolina. This region represents an emerging immigrant destination, where Latino/Hispanic immigrants and refugees constitute 15% of the population, primarily from Mexico (54%) and, also from Honduras, El Salvador, and Guatemala. The community is relatively marginalized, generally living in segregated low-income pockets surrounding the wealthier city centers of Raleigh, Durham, and Chapel Hill. The area witnessed a Latino/Hispanic population shift such that a large segment of the population comprised men who were employed seasonally or through guest worker programs, but in recent years, the community has comprised more women and children indicating that the Latino/Hispanic community has become more established.  Relevant Reference: Tippett, R. (2021). *North Carolina’s Hispanic Community: 2021 Snapshot - North Carolina’s Hispanic population is now greater than one million people*. Carolina Demography. <https://carolinademography.cpc.unc.edu/2021/10/18/north-carolinas-hispanic-community-2021-snapshot/> |

Supplementary Material #2

Semi-Structured Interview Guide

1. For the Latinx immigrants that you treat and those that seek services in this clinic in general, what are the most common problems that they seek services for?
2. In your opinion, what attitudes do you think Latinx immigrants tend to have regarding seeking psychological and psychiatric services?
3. How do you/the clinic most often treat individuals struggling with difficulties associated with adapting to this country? To what extent do these interventions, treatments, and techniques meet the needs of Latinx immigrants seeking services? To what extent do these interventions, treatments, and techniques line up with current guidelines for evidence-based practices?
4. In your opinion, what setting, format, and dose of interventions would be optimal to meet the needs of Latinx immigrants as they are adapting to this country?
5. Do you think there are any evidence-based practices the clinic is not already doing that would represent an added benefit if implemented in the clinic?
6. What supports (or lack thereof) do you think there would be from the clinic regarding implementing additional evidence-based practices? What organizational or community barriers may get in the way of implementing these interventions?

Supplementary Material #3

Description of Qualitative Analysis Process

| Step | Description |
| --- | --- |
| 1. Domain identification | We developed an *a priori* codebook of domains and codes informed by Berry’s (1990; 1997) model of acculturation, Bourhis’(1997) interaction model of acculturation, Cervantes and colleagues’ (2016) framework of facets of acculturative stress, Bronfenbrenner’s Ecological Systems Theory (1992) describing direct and indirect social influences on an individual, and our research team’s prior work pertaining to risk and resilience factors in immigrants and refugees. Our *a priori* codes consisted of both domain codes related to interview questions and category codes; additional category codes that inductively emerged through template analysis were added by consensus based on a review of a gold standard transcript by the coding team (e.g., Moreau et al., 2018). |
| 1. Summary template creation | We created a summary template using the aforementioned domains and categories and tested that template across team members using one interview transcript (Hamilton et al., 2013). During this phase, we developed a matrix with domains and codes, practiced using it on the same transcript, and met to assess feasibility and identify gaps in domains or categories, ensuring consistency across coders. Our team created a matrix with various domains and codes, and all coders practiced using the template on the same transcript. We then met to determine the feasibility of using the template and gaps in domains or categories, as well as to establish consistency in use of the template across coders (Hamilton et al., 2013). We revised domains and categories, creating a comprehensive respondent-by-domain matrix in Excel to be used across all transcripts. We organized domain and category codes by participant without stratifying responses by agency due to the small sample size. |
| 1. Content summarization | Transcripts were divided among coders for content summarization using the matrix. We divided up the transcripts among coders and each summarized the content of the semi-structured interviews using the matrix. Each interview yielded both an audio recording and an analytic memo, from which exemplary quotes were extracted from these sources to support each code Categories and exemplary quotes identified from coding were discussed at weekly meetings with the coding team to establish consistency and resolve any coding differences., with opportunities to resolve any differences in coding. These procedures are consistent with qualitative methods employed in existing implementation research (e.g., Hamilton et al., 2015; Palinkas, 2014). |
| 1. Summary tabulation | We engaged in a “summary tabulation” technique to note frequency counts of each category, in which frequency counts of each category are noted (Abraham et al., 2020). Although we do not present category frequencies, in this paper, we engaged in this process to minimize analyst bias in our interpretation of the prevalence of each code. Tabulations were verified during weekly research team meetings, and parent categories were included in the results of the study if they were discussed by at least two participants. |
| 1. Determine saturation | We retained one final interview to be coded at the end to ensure saturation and sufficiency of existing codes. |

Relevant References Pertaining to Qualitative Analysis Methods

Abraham, T. H., Finley, E. P., Drummond, K. L., Haro, E. K., Hamilton, A. B., Townsend, J. C., ... & Hudson, T. (2020). A Method for Developing Trustworthiness and Preserving Richness of Qualitative Data During Team-Based Analysis of Large Data Sets. *American Journal of Evaluation*, <https://doi.org/10.1177/1098214019893784>

Berry, J. W. (1990). Acculturation and adaptation: A general framework. In W. H. Holtzman & T. H. Bornemann (Eds.), Mental health of immigrants and refugees (pp. 90-102). Hogg Foundation for Mental Health.

Berry, J. W. (1997). Constructing and expanding a framework: Opportunities for developing acculturation research. *Applied Psychology*, 46(1), 62-68. <https://doi.org/10.1111/j.1464-0597.1997.tb01095.x>

Bronfenbrenner, U. (2005). Ecological systems theory (1992). In U. Bronfenbrenner (Ed.), Making human beings human: Bioecological perspectives on human development (pp. 106-173). Sage Publications Ltd.

Bourhis, R. Y., Moise, L. C., Perreault, S., & Senecal, S. (1997). Towards an interactive acculturation model: A social psychological approach. *International Journal of Psychology*, 32(6), 369-386. <https://doi.org/10.1080/002075997400629>

Hamilton, A. (2013). Qualitative methods in rapid turn-around health services research [Cyberseminar]. U.S. Department of Veterans Affairs, Health Services Research & Development. Retrieved from <https://www.hsrd.research.va.gov/for_researchers/cyber_seminars/archives/780-notes.pdf>

Moreau, J. L., Cordasco, K. M., Young, A. S., Oishi, S. M., Rose, D. E., Canelo, I., ... & Hamilton, A. B. (2018). The use of telemental health to meet the mental health needs of women using department of veterans affairs services. *Women's Health Issues*, 28(2), 181-187. <https://doi.org/10.1016/j.whi.2017.12.005>

Palinkas, L. A. (2014). Qualitative and mixed methods in mental health services and implementation research. *Journal of Clinical Child & Adolescent Psychology*, 43(6), 851-861. <https://doi.org/10.1080/15374416.2014.910791>

Supplementary Material #4

Description of Qualitative Rigor

| Qualitative rigor category | Description |
| --- | --- |
| Credibility | Credibility was established through employing multiple coders, discussing coding at weekly research team meetings, and obtaining consensus before adding emergent codes. We have also provided rich quotations to support codes, along with the context of our data collection and their demographic characteristics in aggregate, for readers to determine the transferability of our findings across different contexts. |
| Verification | Verification was established through interviewer-created analytic memos, and maintenance of all iterations of revised codebooks. |
| Reflexivity | We aimed to be reflexive during the research process by having frequent discussions within our racially and ethnically diverse research team, though we are cognizant of our own potential biases, given our collective interest in improving the mental health of immigrants and refugees from Latin America. Interviews were conducted by the PI/interviewer, a clinical psychologist with a Latin American immigrant family background and thus was an “insider” in different ways, having intimate professional and personal knowledge of the topics discussed during the interviews. To formalize the reflection process, she wrote an analytic memo following interviews containing personal thoughts and reactions to the content and process of the interviews.  Our team was multidisciplinary and diverse with regards to racial/ethnic background, training, professional and lived experiences, and gender. Through the coding process, the research team regularly had open discussions pertaining to assumptions they made about participants promoting self-awareness and ability to identify potential sources of bias and therefore enhance the credibility of findings. |

Supplementary Material #5

Description of Qualitative Findings Pertaining to Presenting Problems

| Domain | Category | Description |
| --- | --- | --- |
| Presenting Problems | Mood and bipolar disorders | Any discussion related to a disruption in mood/mood disorder symptoms (e.g. depressed mood, guilt/hopelessness, trouble sleeping, trouble eating, thoughts of self-harm, mania, hypomania) or a formal diagnosis (e.g., major depressive disorder, dysthymia/persistent depressive disorder, bipolar disorder). |
|  | Anxiety disorders | Any discussion related to anxiety symptoms (e.g., muscle tension, worry, panic attacks) or an anxiety disorder (e.g. generalized anxiety disorder, panic disorder, phobias) |
|  | Somatic symptoms | Any discussion related to physical symptoms or sensations experienced by an individual (e.g., headaches, back pain, stomachache). |
|  | Trauma-related disorders | Any discussion related to responses to exposure to trauma (e.g., adverse childhood events), trauma-related symptoms (e.g. hypervigilance, flashbacks, nightmares) or a trauma-related disorder (e.g., PTSD, acute stress disorder). |
|  | Behavioral concerns in school-aged children or youth | Any discussion related to behavioral concerns in school-aged children/youth, including inattention/hyperactivity (e.g., ADHD), oppositional behavior, or generally “acting up” |

Supplementary Material #6

Description of Qualitative Findings Pertaining to the Context in which Therapists Deliver Care to Address Acculturative Stress

| Domain | Category | Description |
| --- | --- | --- |
| Contextual factors | Cultural attitudes and beliefs about mental health problems and treatment | Any discussion related to attitudes (or belief about Western medicine) as being the reason why a service was not used or less likely to be used. For example, this could involve mention about stigma or not perceiving the service would be necessary. |
|  | Access to mental health care | Any discussion related to availability (of the service itself or the providers that facilitate the service) or access (how inaccessible a service is) as being the reason why a service was not used or less likely to be used—like the availability of providers, service is limited to a certain amount of beneficiaries, can only be reached online (for those patients who do not have access to internet) |
|  | Therapist-client match/mismatch | Match refers to any discussion related to both client and provider sharing similar characteristics (that feel salient and important). Mismatch refers to any discussion related to both client and provider sharing similar characteristics (that feel salient and important), such as between the explanatory model or service, or pertaining to client belief that the service could be helpful. |
|  | Trust/mistrust | Trust refers to any discussion related to trust (for the service itself, those providing the service, or the institution associated with providing the service) as being the reason why a service was used or more likely to be used—like trust in the service, providers, or institution. Mistrust refers to any discussion related to mistrust (for the service itself, those providing the service, or the institution associated with providing the service) as being the reason why a service was not used or less likely to be used—like mistrust towards the doctors, the service itself, the institution providing the service, or towards the government for having access to their information. |

Supplement #7

Description of Qualitative Findings Pertaining to Therapeutic Approaches Utilized by Therapists

| Domain | Category | Description |
| --- | --- | --- |
| Theoretical Orientation | Cognitive Behavioral Treatments | Any discussion related to theories that provide a framework for mental health care providers to interpret a patient's behavior, thoughts, and feelings and help them navigate a patient's journey from diagnosis to post-treatment.  Any discussion related to the approach whose focus is on cognitions and behaviors. |
| Settings | Outpatient care | Any discussion related to delivery of psychosocial intervention to individuals who primarily reside outside of the facility providing treatment (e.g., seeing a therapist once a week). |
|  | Community settings | Any discussion related to participating in programming is provided through community centers or agencies, with the goal of acculturating or managing acculturative stress. |
| Frequency | --- | Any discussion related to the frequency with which services are delivered – like weekly, biweekly, or monthly. |
| Format | Individual | Any discussion related to delivery of a psychosocial intervention from one therapist to at least one patient. An individual is the identified patient. |
|  | Group | Any discussion related to delivery of a psychosocial intervention from one therapist to several patients (at least more than one). An individual is the identified patient, but in a group. |
| Components | Validation and normalizing | Any discussion related to allowing another person to discuss their thoughts and feelings in a safe environment. May encompass providing warmth, encouragement, compliments, etc. May also include recognition or affirmation that a person, their thoughts, and feelings or opinions are valid or worthwhile. |
|  | Psychoeducation | Any discussion related to the process of providing education and information to those seeking or receiving mental health services, such as people diagnosed with mental health conditions (or life-threatening/terminal illnesses) and their family members. |
|  | Discussion, reflection, and drawing out narratives | Any discussion related to an intervention that incorporates discussion, reflection, or sharing one’s narrative (especially when related to the process of immigration and/or adapting to a new environment). |
|  | Perspective taking/ cultural brokering | Any discussion related to how providers engage client/patient(s) in considering another person’s perspective or to navigate between cultures. |
|  | Connection to community resources | Any discussion related to elements of an intervention that are focused on providing participants with information about community resources and/or connecting them with those resources. |
|  | Therapist self-disclosure | Any discussion related to the process of communication by which the provider reveals information about themself to another. The information can be descriptive or evaluative, and can include thoughts, feelings, aspirations, goals, failures, successes, fears, and dreams, as well as one's likes, dislikes, and favorites. |
|  | Behavioral strategies | Any discussion related to strategies aimed at managing or modifying a participant's behavior (e.g., goal setting, strategies/plans to decrease harmful or risky behaviors). |
